# Transgenic Mice Overexpressing Renin Exhibit Glucose Intolerance and Diet-Genotype Interactions

**DOI:** 10.3389/fendo.2012.00166

**Published:** 2013-01-07

**Authors:** Sarah J. Fletcher, Nishan S. Kalupahana, Morvarid Soltani-Bejnood, Jung Han Kim, Arnold M. Saxton, David H. Wasserman, Bart De Taeye, Brynn H. Voy, Annie Quignard-Boulange, Naima Moustaid-Moussa

**Affiliations:** ^1^Genome Science and Technology Program, University of TennesseeKnoxville, TN, USA; ^2^Department of Physiology, Faculty of Medicine, University of PeradeniyaPeradeniya, Sri Lanka; ^3^Pellissippi StateKnoxville, TN, USA; ^4^Department of Pharmacology, Physiology and Toxicology, School of Medicine, Marshall UniversityHuntington, WV, USA; ^5^Department of Animal Science, University of TennesseeKnoxville, TN, USA; ^6^Department of Molecular Physiology and Biophysics, School of Medicine and Mouse Metabolic Phenotyping Center, Vanderbilt UniversityNashville, TN, USA; ^7^INRA-AgroParisTech UMR 914Paris, France; ^8^Nutritional Sciences, Texas Tech UniversityLubbock, TX, USA

**Keywords:** adipose tissue, renin-angiotensin system, insulin resistance, angiotensin II

## Abstract

Numerous animal and clinical investigations have pointed to a potential role of the renin-angiotensin system (RAS) in the development of insulin resistance and diabetes in conditions of expanded fat mass. However, the mechanisms underlying this association remain unclear. We used a transgenic mouse model overexpressing renin in the liver (RenTgMK) to examine the effects of chronic activation of RAS on adiposity and insulin sensitivity. Hepatic overexpression of renin resulted in constitutively elevated plasma angiotensin II (four- to six-fold increase vs. wild-type, WT). Surprisingly, RenTgMK mice developed glucose intolerance despite low levels of adiposity and insulinemia. The transgenics also had lower plasma triglyceride levels. Glucose intolerance in transgenic mice fed a low-fat diet was comparable to that observed in high-fat fed WT mice. These studies demonstrate that overexpression of renin and associated hyperangiotensinemia impair glucose tolerance in a diet-dependent manner and further support a consistent role of RAS in the pathogenesis of diabetes and insulin resistance, independent of changes in fat mass.

## Introduction

The renin-angiotensin system (RAS) plays an important role in the regulation of blood pressure, fluid, and electrolyte balance (Schmieder et al., 2007). Angiotensinogen (AGT), the precursor peptide of this system, undergoes successive enzymatic cleavages by renin and angiotensin converting enzyme (ACE) to yield angiotensin I (Ang I) and angiotensin II (Ang II) respectively. The latter is the main bioactive peptide of this system, which acts via two G-protein coupled receptors, namely angiotensin Type-1 (AT1) and Type-2 (AT2) receptors, to exert its physiological effects. Because AT1 activation by Ang II leads to elevation of blood pressure, ACE inhibitors (ACEI) and AT1 blockers (ARB) are pharmacologically used as anti-hypertensive agents (Schmieder et al., 2007).

Interestingly, several clinical studies have shown that patients on RAS blockers have a lower risk of developing Type-2 diabetes when compared to patients on other anti-hypertensive medications (Vermes et al., 2003; Bosch et al., 2006). Moreover, RAS blockade prevents and reverses insulin resistance induced by high-fat feeding in rodents (Lee et al., 2008). Given that plasma and tissue levels of several RAS components positively correlate with body mass index (Schorr et al., 1998; Van Harmelen et al., 2000), it is possible that overactivation of the RAS is linked to the pathogenesis of insulin resistance in obesity. Indeed, genetic deletion of AGT, ACE, renin, AT1, or AT2 protects rodents from diet-induced obesity and insulin resistance (Massiera et al., 2001b; Yvan-Charvet et al., 2005; Takahashi et al., 2007; Jayasooriya et al., 2008). Conversely, chronic RAS overactivation via Ang II infusion (Ogihara et al., 2002) leads to glucose intolerance and insulin resistance in rodents, further supporting a role of RAS overactivation in the pathogenesis of insulin resistance.

Although obesity and increased adiposity are associated with RAS overactivation, it is not clear whether systemic RAS overactivation can lead to both obesity and insulin resistance. It is important to test this because studies have documented differences in RAS activity in humans, which have been attributed to polymorphisms in RAS coding (Jeunemaitre et al., 1999; Jeunemaitre, 2008) or promoter regions (Xiao et al., 2006). Therefore, understanding the implications of chronic elevation of RAS may help provide insight into metabolic consequences of chronically elevated RAS in humans.

While overexpression of RAS is consistently associated with insulin resistance and glucose intolerance, the effect of chronic RAS overactivation on adiposity is not clear. This is further complicated by existence of local RAS in several tissues with the local effects complicating the understanding of systemic effects of RAS (Kalupahana and Moustaid-Moussa, 2012b). For example, overexpression of AGT in adipose tissue increases adiposity and blood pressure and leads to insulin resistance (Massiera et al., 2001a; Kalupahana et al., 2012). However, acute or chronic systemic RAS overactivation leads to decreased fat mass despite the development of insulin resistance (Brink et al., 1996). This suggests that increased fat mass in the case of adipose RAS overexpression may be due to local effects of Ang II production within adipose tissue.

To further dissect effects of elevated systemic Ang II on insulin sensitivity and adiposity, we used a unique mouse model in which Ang II is chronically elevated throughout life time through genetic manipulation. This mouse model is a unique genetic minipump model in which renin is overexpressed in the liver. Given that renin release is the rate-limiting step in the systemic RAS, this model offers the advantage of constant renin overexpression independent of homeostatic control and a lifelong elevated level of Ang II. As expected, these transgenic mice (RenTgMK; Caron et al., 2002) exhibit elevated levels of circulating renin and Ang I and develop chronic hypertension along with other pathological manifestations (Caron et al., 2002, 2004). The RenTgMK mice thus allow us also to study the effects of systemic chronic elevations of Ang II on adiposity and glucose homeostasis, so that we can dissect the effects of systemic vs. adipose RAS by comparing these results with the ones previously reported for local adipose overexpression of RAS (Massiera et al., 2001a).

We report here that elevated circulating Ang II due to renin overexpression leads to glucose intolerance, which is further exacerbated by high-fat feeding. Unexpectedly, these mice exhibit otherwise normal glucose metabolism and a transgene dose-dependent decrease in fat mass and insulinemia.

## Materials and Methods

### Animals

RenTgMK transgenic mice were kindly provided by Dr. Oliver Smithies, University of North Carolina, Chapel Hill, NC, USA (Hatada et al., 1999). Briefly, a renin transgene consisting of portions of the Ren2 and Ren-1d genes (Ren2/1d) was inserted into the genome at the ApoA1/ApoC3 locus via homologous recombination and placed under control of an albumin promoter/enhancer (AlbP/E) to achieve liver-specific expression.

Male heterozygous RenTgMK (RenTgMK^−/+^) mice on an isogenic SvEv 129/6 background were crossed with wild-type (WT) SvEv females. Subsequent heterozygous F1 progeny were mated to generate the F2 offspring that were used in this study. Mice used in this study were bred and maintained at the University of Tennessee accredited animal facility, on a 12h:12h light-dark cycle at 22°C and fed a standard rodent chow and water *ad libitum*. All experiments were approved by the Institutional Animal Care and Use Committee at the University of Tennessee.

### Genotyping

DNA was extracted from tail tips as previously described (Truett et al., 2000). PCR-based genotyping was performed using three primers: p1, 5′-TGGGATTCTAACCCTGAGGACC-3′; p2, 5′-CACAGATTGTAACTGCAAATCTGTCG-3′; p3, 5′-GTTCTTCTGAGGGGATC-GGC-3′ (Sigma Genosys, The Woodlands, TX, USA) as previously described (Caron et al., 2002).

### Glucose tolerance test

Mice were fasted overnight prior to the glucose tolerance test (GTT). Blood was collected in heparinized capillary tubes from the orbital sinus prior to intra-peritoneal injection with glucose (1 g/kg body weight), and then 15, 30, 60, 90, and 120 min after injection. Plasma glucose concentrations were calculated using a One Touch ultra-monitoring system (Johnson & Johnson, Co., New Brunswick, NJ, USA). The GTT was performed on mice 10 weeks old and repeated when the mice reached 20 weeks of age and the area under the curve (AUC) for glucose and insulin were calculated.

### Plasma measurements

Serum was separated from blood samples collected during the GTT by centrifugation at 3000 rpm for 15 min at 4°C and then stored in aliquots at −80°C until assayed. Serum insulin, leptin, and adiponectin levels were measured in duplicate using commercially available ELISA kits following the manufacturer’s protocol (insulin cat# 90060 and leptin cat# 90030, Crystal Chem, Inc., Downers Grove, IL, USA; adiponectin cat# EZMADP-60, Linco Research, Billerica, MA, USA). Absorbance was read at 450 nm on a Packard SpectraCount microplate reader (Packard Instrument, Co., Meriden, CT, USA).

### Diet study

Male heterozygous (RenTgMK^+/−^) mice and their WT littermates were randomly assigned to either a high-fat diet (60% kcal from fat cat# D12492, Research Diets, Inc., New Brunswick, NJ, USA) or a low-fat diet (10% kcal from fat cat# D12450B, Research Diets, Inc., New Brunswick, NJ, USA) for 18 weeks. Each diet group (*n* = 6/group) was comprised of three male RenTgMK^+/−^ mice and three male WT mice. Body weight measurements were acquired weekly for the duration of the study. At the conclusion of the 18-week diet study, a GTT was performed and plasma insulin, leptin, and adiponectin concentrations were measured, as described above. Mice were sacrificed 1 week after the GTT.

### Metabolic studies

Metabolic studies of the RenTgMK mice were performed at the Mouse Metabolic Phenotyping Center (MMPC) at Vanderbilt University, Nashville, TN, USA. Glucose infiltration rate, glucose turnover rate, endogenous glucose turnover rate, and clearance were measured. Whole-body insulin activity *in vivo* was examined via euglycemic hyperinsulinemic clamp. Detailed procedure has been previously reported (Ayala et al., 2006). Briefly, to assess insulin sensitivity and glucose metabolism, insulin was continuously administered via euglycemic hyperinsulinemic clamp. Catheters were chronically implanted in the jugular vein and carotid artery. Arterial glucose levels were measured every 5–10 min during 120 min and glucose infusion rates were determined based on the arterial glucose measurements. Plasma glucose turnover was measured in RenTgMK^+/−^ and WT males (*n* = 8–12/group). Mice were continuously infused with [3-^3^H]glucose at a rate of 0.4 μCi/min. Glucose appearance (Ra) and disappearance (Rd) rates were estimated as the ratio of the rate of infusion of [3-^3^H]glucose and the steady-state plasma [^3^H]glucose specific activity (dpm/mg), and the glucose disappearance was assumed to be equal to the steady-state Ra rate. Glucose clearance was calculated by dividing the Rd by the arterial glucose concentration. To measure tissue-specific glucose uptake, mice were injected with 12 μCi of [^3^H]-labeled 2-deoxyglucose ([2-^3^H] DG). Arterial plasma samples were collected in intervals for 40 min before mice were anesthetized and tissues were extracted and frozen in liquid nitrogen until further analysis.

### Pancreas histology and immunostaining

The pancreas was collected from WT and transgenic mice. Tissues for immunohistochemistry were fixed in 10% neutral, phosphate-buffered formalin for 24 h and paraffin-embedded. Subsequently, the paraffin-embedded tissues were processed in 4-μm sections. Sections were stained using rabbit anti-glucagon polyclonal antibody and guinea pig anti-insulin serum (both from Millipore, Billerica, MA, USA). For fluorescence detection, goat anti-guinea pig IgG coupled to Texas Red and donkey anti-rabbit IgG coupled to Cy3 were used (both from Jackson ImmunoResearch, West Grove, PA, USA) followed by Vectashield Mounting Medium with 4,6-diamidino-2-phenylindole (Vector Laboratories, Burlingame, CA, USA) for nuclear staining.

### Statistical analysis

Data were analyzed in SAS (SAS Institute, Inc., Cary, NC, USA) using a mixed model analysis of variance (http://dawg.utk.edu). Fisher’s test followed by Tukey’s *post hoc* test was used for mean separation. *P* < 0.05 was considered statistically significant. Data are reported as the means ± SE.

## Results

### Body weight, fat pad weight, and metabolic parameters

**Figure 1:**
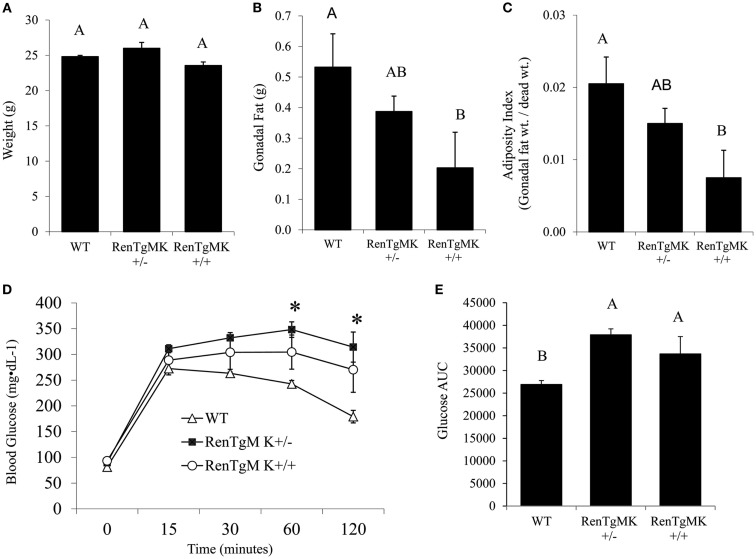
**Body and fat pad weight and glucose tolerance in male RenTgMK mice**. **(A)** Body weight at the age of 20 weeks. **(B)** Mice were sacrificed at the end of week 20 and gonadal fat pads were collected and weighed. **(C)** The adiposity index was determined by dividing gonadal fat pad weight by final body weight. A glucose tolerance test (GTT) was administered after overnight fasting. **(D)** Blood glucose levels were measured at 0, 15, 30, 90, and 120 min and plotted on a graph. **(E)** Area Under the Curve (AUC) was calculated as described in the experimental procedures. Values are means ± SE. *n* = 6 For WT; *n* = 5 for RenTgMK^+/−^; *n* = 4 for RenTgMK^+/+^. Different letters indicate a significant difference (*P* < 0.05). *Significantly different (*P* < 0.05) from WT.

Body weights were comparable between mice with either one or two copies of the renin transgene and WT control mice (Figure 1A). Gonadal fat pad weight (Figure 1B) and adiposity index (gonadal fat pad weight divided by body weight; Figure 1C) were significantly lower in homozygous mice compared to WT littermates (*P* < 0.05). Fasting serum glucose, leptin, and adiponectin levels were comparable between all genotypes (Table 1). Fasting serum insulin, however, was significantly lower in the transgenic mice (both homozygous and heterozygous) compared to WT littermates (*P* < 0.05). Serum C-peptide levels, on the other hand, were higher in the transgenics than in the WT mice (Table 1). Interestingly, serum triglycerides were significantly lower in the homozygous mice compared to WT littermates. On this low-fat chow diet condition, the overall metabolic phenotype was less pronounced in female transgenic mice compared to WT female littermates (data not shown).

**Table 1:** **Serum metabolic markers in male wild-type and RenTgMK mice**.

	WT	RenTgMK^+/−^	RenTgMK^+/+^	*P* value
Glucose, mg/dl	81.2 ± 6.4	89.8 ± 3.2	93.3 ± 5.1	0.292
Insulin, ng/ml	0.62 ± 0.07^a^	0.42 ± 0.05^b^	0.36 ± 0.07^b^	**0.033**
Leptin, ng/ml	2.1 ± 0.7	2.3 ± 0.6	1.3 ± 0.5	0.535
Adiponectin, μg/ml	8.7 ± 1.4	10.8 ± 2.5	7.6 ± 0.9	0.479
C-peptide, ng/ml	1.4 ± 0.1^b^	1.9 ± 0.2^a^	2.0 ± 0.1^a^	**0.007**
FFA, mM	0.84 ± 0.10	0.93 ± 0.06	0.91 ± 0.11	0.791
Triglycerides, mg/dL	60.1 ± 6.4^a^	44.5 ± 8.6^a,b^	23.7 ± 8.7^b^	**0.018**

*Values are means ± SE. Animals were 21 weeks old. Initial body weight measurements were taken at 10 weeks. Blood was collected after fasting overnight and metabolic parameters were measured from serum. *n* = 6 For WT; *n* = 5 for RenTgMK^+/−^; *n* = 4 for RenTgMK^+/+^. C-peptide, connecting peptide; FFA, free fatty acid*.

*Means in a row with superscripts without a common letter differ, *P* < 0.05*.

*Numbers in bold indicate a significance of P<0.05*.

### Glucose intolerance in RenTg mice

To assess glucose tolerance in the RenTg mice, an intra-peritoneal GTT was administered. Baseline fasting glucose levels were comparable between WT, RenTgMK^+/−^ and RenTgMK^+/+^ mice (81.17 ± 15.68, 89.80 ± 7.16, and 93.25 ± 10.28 mg/dl, respectively). Heterozygous mice maintained significantly higher levels of glycemia compared to WT within 60 min and remained elevated throughout the GTT (Figure 1D). These differences were observed as early as 10 weeks of age (data not shown) and became more pronounced with age by 20 weeks. Glucose intolerance in male RenTgMK mice was also evident from a comparison of the glucose AUC (Figure 1E). The AUC values for both heterozygous and homozygous mice were higher (*P* < 0.05) than that of WT mice implying greater glucose intolerance in the transgenics. In females, no significant differences in GTT were observed between the three genotypes at 20 weeks of age in these low-fat feeding conditions (data not shown).

### Metabolic phenotyping of RenTg mice

**Figure 2:**
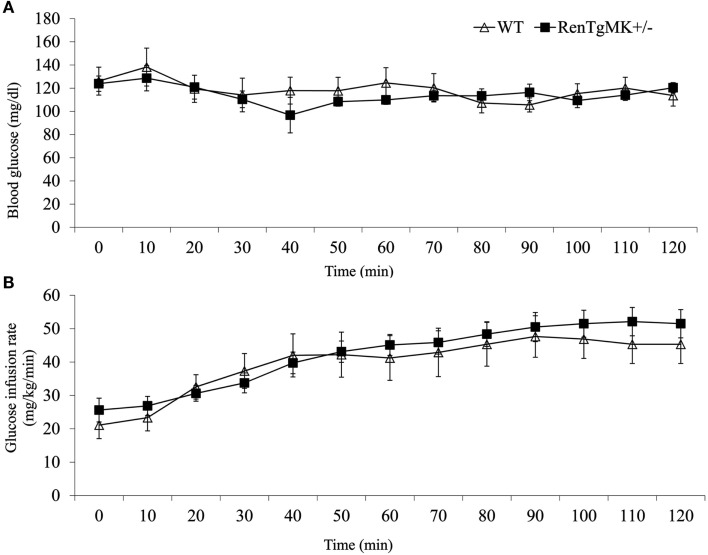
**Assessment of insulin sensitivity in male wild-type and RenTgMK^+/−^ mice using hyperinsulinemic euglycemic clamp**. Changes in blood glucose concentration **(A)** and glucose infusion rate **(B)** over time are shown. Values are means ± SE. Animals were approximately 9 months old. *n* = 8–9 For WT; *n* = 7 for RenTgMK^+/−^.

Insulin resistance is commonly associated with high adiposity. The paradoxical glucose intolerance despite low adiposity and low insulinemia in the renin transgenic male mice vs. control littermates led us to further investigate whether these differences were due to altered insulin sensitivity and/or glucose production or utilization in this model. Accordingly, metabolic studies at the NIH MMPC at Vanderbilt University were conducted. Male heterozygous mice were compared to WT mice because males exhibited glucose intolerance and sufficient numbers could be obtained from a few litters. Steady-state glucose infusion rate (Figure 2), overall tissue-specific glucose uptake, glucose metabolism, and endogenous glucose production (Table 2) did not significantly differ between RenTgMK and WT mice, indicating normal insulin sensitivity in the transgenics.

**Table 2:** **Metabolic characteristics and accumulation of [2-^3^H]DG during the hyperinsulinemic-euglycemic clamp experiments in male wild-type and RenTgMK^+/−^ mice**.

	WT	RenTgMK^+/−^	*P* value
**GLUCOSE KINETICS**			
Blood glucose, mg/dl	114.7 ± 5.8	114.6 ± 5.0	0.996
GTR, mg/kg/min	47.4 ± 4.0	49.4 ± 6.7	0.794
endoGTR, mg/kg/min	4.96 ± 4.62	−2.29 ± 7.60	0.416
Glucose clearance, mg/kg/min	42.0 ± 3.1	43.7 ± 5.7	0.790
GIR, mg/kg/min	42.4 ± 4.3	51.7 ± 4.0	0.142
**ACCUMULATION OF [2-^3^H]DG**			
Soleus, μg/min/mg tissue	0.035 ± 0.008	0.036 ± 0.005	0.923
Gastro, μg/min/mg tissue	0.025 ± 0.005	0.033 ± 0.003	0.229
Vastus l., μg/min/mg tissue	0.041 ± 0.007	0.047 ± 0.005	0.550
WAT, μg/min/mg tissue	0.004 ± 0.001	0.006 ± 0.001	0.067
Diaphragm, μg/min/mg tissue	0.131 ± 0.019	0.091 ± 0.007	0.103
Heart, μg/min/mg tissue	0.431 ± 0.057	0.320 ± 0.043	0.161
Brain, μg/min/mg tissue	0.048 ± 0.005	0.049 ± 0.003	0.888

*Values are means ± SE. Animals were approximately 9 months old. *n* = 8–9 For WT; *n* = 7 for RenTgMK^+/−^. GTR, glucose turnover rate; endoGTR, endogenous glucose turnover rate; GIR, glucose infusion rate; [2-^3^H]DG, 2-deoxy-[^3^H]glucose; Gastro, gastrocnemius; Vastus l., vastus lateralis; WAT, white adipose tissue*.

### Effect of high-fat diet on body weight, adiposity, circulating adipokines, and glucose tolerance

As described above, renin transgene overexpression led to impaired glucose tolerance compared to WT mice when mice were fed a low-fat chow diet. To test whether the genetic differences would be exacerbated by high-fat feeding, we fed male heterozygous and WT mice a low- or high-fat diet to investigate diet-gene interactions.

**Figure 3:**
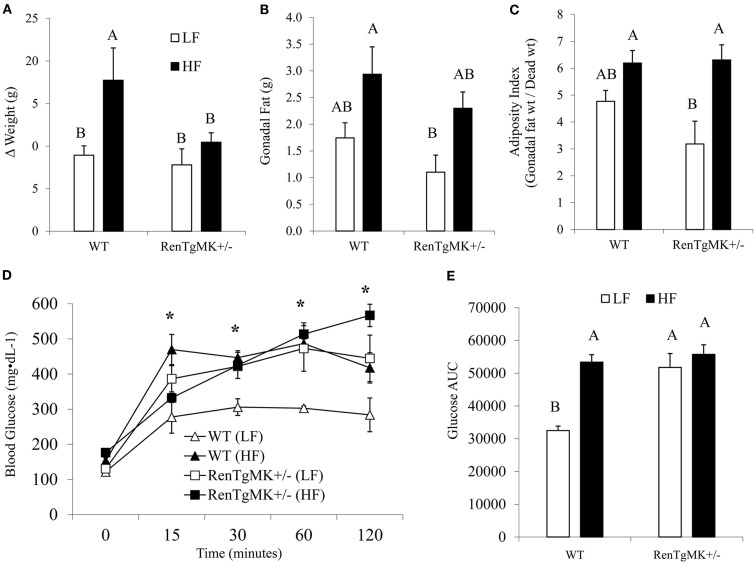
**Effect of high-fat diet on body and fat pad weight and glucose tolerance of male wild-type and RenTgMK^+/−^ mice**. **(A)** Weight gain was calculated as the difference between the initial body weight measured at week 1 and the final weight measured after 18 weeks. **(B)** Mice were sacrificed at the end of week 19 and gonadal fat pads were collected and weighed. **(C)** The adiposity index was determined by dividing gonadal fat pad weight by final body weight X 100. **(D)** A glucose tolerance test (GTT) was administered after overnight fasting. Blood glucose levels were measured at 0, 15, 30, 90, and 120 min and plotted on a graph. **(E)** Area Under the Curve (AUC) was calculated as described in the experimental procedures. Values are means ± SE. *n* = 3 For each group. *Significantly different (*P* < 0.05) from WT-LF. Different letters indicate a significant difference (*P* < 0.05).

Body weights were not significantly different between groups at the start of the randomized diet study (Table 3). High-fat feeding increased body weight only in the wild-type mice (Figure 3A). Mice of both genotypes showed a trend for increased fat pad weight and adiposity with high-fat feeding, although the difference was only significant for adiposity in the RenTgMK^+/−^ mice (Figures 3B,C).

**Table 3:** **Effects of high-fat diet on body weight and metabolic characteristics in male wild-type and RenTgMK^+/−^ mice**.

	WT		RenTgMK^+/−^		*P* value		
	LF	HF	LF	HF	Geno	Diet	Geno X diet
Initial body weight, g	27.2 ± 2.2	29.2 ± 1.4	26.2 ± 0.6	25.7 ± 0.9	NS	NS	NS
Final body weight, g	37.6 ± 3.2^a,b^	46.9 ± 5.6^a^	35.4 ± 1.8^b^	37.8 ± 2.1^a,b^	NS	NS	NS
Glucose, mg/dL	92.3 ± 3.2^b^	123.7 ± 7.7^a^	98.3 ± 5.2^b^	127.3 ± 2.9^a^	NS	**0.001**	NS
Insulin, ng/ml	1.29 ± 0.31	2.07 ± 0.60	0.91 ± 0.04	1.14 ± 0.25	NS	NS	NS
Leptin, pg/ml	9.5 ± 1.6	16.2 ± 2.9	7.6 ± 1.1	12.7 ± 2.3	NS	**0.019**	NS
Adiponectin, μg/ml	12.6 ± 0.3	14.1 ± 2.0	12.9 ± 0.4	12.4 ± 0.2	NS	NS	NS
Resistin, pg/ml	475.8 ± 37.3^b^	702.2 ± 19.0^a^	545.8 ± 48.7^a,b^	493.3 ± 83.9^b^	NS	NS	**0.030**
MCP-1, pg/ml	29.9 ± 8.0	53.0 ± 18.9	30.5 ± 1.6	34.0 ± 12.9	NS	NS	NS
PAI-1, pg/ml	3903.4 ± 666.2	5903.4 ± 834.8	4733.5 ± 734.0	4526.8 ± 961.3	NS	NS	NS
C-peptide, ng/ml	1.9 ± 0.3	3.3 ± 1.2	2.4 ± 0.4	2.3 ± 0.2	NS	NS	NS
FFA, mM	1.00 ± 0.18	0.80 ± 0.04	1.23 ± 0.36	1.16 ± 0.39	NS	NS	NS
Triglycerides, mg/dL	132.4 ± 16.5^a^	66.9 ± 4.1^b^	66.8 ± 4.4^b^	39.2 ± 5.4^b^	**0.001**	**0.001**	**0.074**

*Values are means ± SE. Animals were fed a high-fat or low-fat diet for 19 weeks. Initial body weight measurements were taken at the beginning of the study. Mice were 3–5 months old. Blood was collected after fasting overnight and metabolic parameters were measured from serum. *n* = 3 For each group. LF, low-fat; HF, high-fat; MCP-1, monocyte chemoattractant protein-1; PAI-1, plasminogen activator inhibitor-1; C-peptide, connecting peptide; FFA, free fatty acid*.

*Means in a row with superscripts without a common letter differ, *P* < 0.05*.

*Numbers in bold indicate a significance of P<0.05*.

Changes in adiposity are known to alter hormonal and metabolite levels. As expected, high-fat feeding increased serum glucose and leptin levels in both male WT and transgenic mice (*P* < 0.05 for diet effect – Table 3). Interestingly, high-fat feeding also increased serum resistin levels in WT, but not in transgenic males (Table 3). In the WT males, serum triglyceride concentration was higher in the low-fat fed mice when compared to high-fat fed ones (Table 3). This effect was minimal in the transgenics.

Low-fat fed male heterozygous mice exhibited a higher glucose excursion and area under the glucose curve compared to their WT counterparts, indicating glucose intolerance (Figures 3D,E). High-fat feeding did not exacerbate glucose intolerance in RenTgMK mice.

### Pancreas histology and immunostaining of RenTg mice

**Figure 4:**
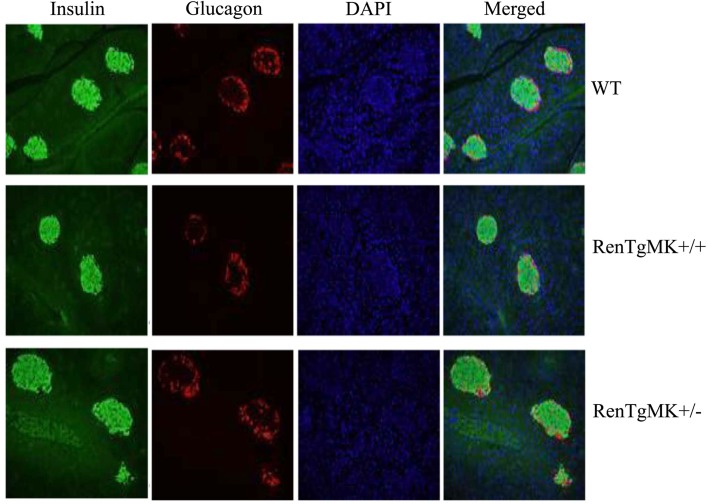
**Islet pathology**. Pancreas histology and immunostaining were conducted to assess islet morphology and hormone content in male wild-type and RenTgMK mice, 20 weeks of age.

Because of the consistently lower insulin levels in heterozygous mice compared to WT mice, we performed immunohistological studies in the pancreas to assess islet morphology and hormone content. In both genotypes, islets appeared normal and exhibited comparable staining for glucagon and insulin (Figure 4).

## Discussion

Many lines of evidence have linked activation of the RAS to the development of obesity and insulin resistance (Schorr et al., 1998; Van Harmelen et al., 2000), but the effects of increased circulating levels of angiotensins on adiposity remain controversial. We hypothesized that chronic systemic RAS activation via transgenic renin overexpression in the liver would lead to glucose intolerance and systemic insulin resistance. We also predicted that increased systemic Ang II would increase adiposity, based on previous work by us and others showing that Ang II increases adipocyte lipogenesis and triglyceride storage. Our results demonstrate that elevated circulating Ang II due to renin overexpression leads to glucose intolerance, but with consistently lower levels of plasma insulin. Further, chronic elevation of systemic Ang II by hepatic overexpression of the renin gene led to a reduction rather than an increase in adiposity in male mice. However, these mice exhibit otherwise normal glucose metabolism and a transgene dose-dependent decrease in insulinemia.

### Glucose intolerance in RenTgMK mice

Consistent with previous studies of Ang II infusion and transgenic renin expression (Lee et al., 1996), male RenTgMK transgenic mice exhibited glucose intolerance, even on a low-fat diet. However, despite this glucose intolerance, the RenTgMK mice maintained low fasting insulinemia and normal insulin sensitivity, as indicated by normal steady-state glucose infusion during the hyperinsulinemic, euglycemic clamp studies. RenTgMK mice maintained low insulinemia even under high-fat feeding. The glucose intolerance in RenTgMK mice in the presence of normal fasting glucose levels and low insulinemia, a feature that is a rather typical hallmark of increased insulin sensitivity, could be due to decreased insulin production/secretion and/or increased insulin clearance. Serum C-peptide level was higher in heterozygous compared to WT mice arguing against decreased insulin secretion accounting for low insulinemia in the RenTgMK mice. Immunohistochemistry of the pancreas indicated normal islet morphology and hormone content, possibly indicating normal pancreatic function. However, such studies are only qualitative and do not allow to detect clear quantitative differences. Thus, it is probably insulin clearance, rather than insulin secretion that may be altered in this model.

Liver is the primary site of insulin clearance (Duckworth et al., 1998), which can be affected by both nutritional and hormonal signals. Insulin clearance rate is heritable (Goodarzi et al., 2005) and is reduced in obesity and Type-2 diabetes (Duckworth et al., 1998). Therefore, it could be an important factor in the pathogenesis of Type-2 diabetes. Conversely, there are mouse models which exhibit increased insulin clearance such as the mouse overexpressing carcinoembryonic antigen-related cell adhesion molecule 1 (CEACAM1) in the liver (Najjar, 2002). Additional studies beyond the scope of this work will be required to address whether the RAS is involved in regulating insulin clearance.

The finding that the glucose intolerance in male transgenic mice did not worsen with high-fat feeding could possibly indicate that RAS overactivation could at least in part play a role in high-fat diet-induced obesity. Along the same lines, mice overexpressing AGT in adipose tissue also develop glucose intolerance on a low-fat diet, which is not further exacerbated by high-fat feeding (Kalupahana et al., 2012). Female transgenic mice exhibited normal glucose tolerance on a low-fat diet while males became glucose intolerant on the same diet when compared to WT littermates. Further, female transgenics became glucose intolerant when fed a high-fat diet (data not shown).

It is likely that the metabolic phenotype of the RenTgMK mice is due to Ang II effects, rather than the effects of renin acting on the renin/prorenin receptor. We argue this because in renin knockout mice, the metabolic phenotype of increased insulin sensitivity and resistance to high-fat diet-induced glucose intolerance and insulin resistance was reversed by Ang II infusion (Takahashi et al., 2007). It is also likely that these effects are mediated via angiotensin receptors, as previous studies on the RenTgMK mice demonstrated that AT1 receptor blockade reversed renal pathology and normalized blood pressure in the RenTgMK mice (Caron et al., 2002). Alternative mechanisms may involve direct effects of renin mediated by the renin/prorenin receptor on the vasculature or adipose tissue. Indeed, renin receptors are expressed in adipose tissue (Achard et al., 2007) and therefore may mediate the observed adipose tissue phenotype.

### RAS overactivation and insulin resistance

Renin-angiotensin system overactivation via chronic Ang II infusion leads to the development of systemic insulin resistance in rodents. This is, in most part, due to the Ang II-mediated impairment of skeletal muscle glucose transport and utilization (Kalupahana and Moustaid-Moussa, 2012a). Ang II impedes the insulin-mediated tyrosine phosphorylation of the insulin receptor substrate (IRS)-1, activation of Akt, and translocation of glucose transporter (Glut)-4 in the skeletal muscle in an NADPH oxidase, AT1, and NF-kB-dependent manner. Ang II also increases hepatic glucose production, which also potentially contributes to altered systemic insulin sensitivity. In contrast, the RenTgMK mice in this study exhibited normal systemic insulin sensitivity. While the exact underlying mechanisms for this discrepancy of insulin sensitivity between different models of RAS overactivation are unknown, it is possible that the low insulinemia present in the RenTgMK mice could protect these mice from the development of insulin resistance. Previous studies have shown that an increase in plasma insulin by itself can induce insulin resistance. In the study by Shanik et al. (2008), mice transfected with extra copies of the insulin gene had a two- to four-fold increase in plasma insulin and exhibited normal body weight, insulin resistance and hypertriglyceridemia.

Unlike models of chronic Ang II infusion (Ran et al., 2004), RenTgMK mice exhibited lower plasma triglyceride levels. Thus, the hypoinsulinemia in the RenTgMK could also potentially explain the low serum triglyceride levels seen in these mice. Given this metabolic phenotype of RenTgMK mice, it would be interesting to explore whether the insulin resistance seen in several models of chronic RAS overactivation is insulin-dependent and further studies are warranted. The issue of whether the renin receptor may also in part modulate insulin sensitivity merits further investigation as well.

### RAS overactivation and adiposity

Both human and rodent studies have shown that obesity and increased adiposity are associated with both systemic and adipose RAS overactivation (Kalupahana and Moustaid-Moussa, 2012a). However, it is not known whether primary RAS overactivation leads to obesity. Transgenic mouse models clearly demonstrate that manipulating components of the RAS alters adiposity: mice overexpressing AGT in adipose tissue have increased adiposity, while deletion of either the AGT or Ang II receptor genes reduces fatness. Paradoxically, previous studies of chronic Ang II infusion in rodents have shown that chronic systemic RAS overactivation leads to weight loss, rather than weight gain (Griffin et al., 1991; Cassis et al., 1998). The transgenic TGR(mREN2)27 rat overexpressing the mouse Ren2 renin gene also has a lean phenotype (Mullins et al., 1990; Langheinrich et al., 1996; Lee et al., 1996). Similar to these findings, the RenTgMK mice also exhibited lower fat mass compared to WT littermates. The adipose mass was significantly decreased by the renin transgene in a gene dosage-dependent manner. In contrast, mice with primary AGT overproduction in adipose tissue exhibit higher adiposity (Massiera et al., 2001a). Further, deletion of AGT and other RAS genes leads to lower fat mass and resistance to diet-induced obesity (Massiera et al., 2001b; Takahashi et al., 2007). Thus, it appears that while systemic RAS overactivation leads to reductions in body weight, local increases in RAS activity in adipose tissue leads to increased adiposity.

The low-fat mass observed following Ang II infusion is attributed to both increased energy expenditure and reduced energy intake (Brink et al., 1996; Cassis et al., 1998). In the RenTgMK mouse model, we did not detect any significant differences in food intake (data not shown). Activation of the sympathetic nervous system may also account for changes in weight via modulation of lipid metabolism and energy expenditure by catecholamines (Cassis, 2000). The differential effect of systemic vs. adipose specific RAS overactivation on adiposity indicates that specific local overproduction of AGT in adipose tissue *per se*, may be required for increasing adiposity. Indeed, Ang II exerts local anabolic effects in the adipose tissue (Massiera et al., 2001a). Ang II also increases lipogenic gene expression and enzyme activity in 3T3-L1 murine adipocytes and human adipocytes *in vitro* (Jones et al., 1997). This is also in agreement with studies showing differentiation-dependent increase in AGT gene expression and secretion in preadipocytes (Kim and Moustaid-Moussa, 2000). Ubiquitous inactivation of AGT, on the other hand, results in significant loss of fat mass. However, it is unclear whether targeted inactivation of AGT in adipose tissue would specifically alter fat mass and such studies would convincingly confirm the role of adipose AGT in modulating insulin resistance or fat mass.

In summary, our data demonstrate that transgenic hepatic overexpression of renin leads to glucose intolerance, decreased fat mass, hypoinsulinemia, and hypotriglyceridemia, with normal systemic insulin sensitivity. The hypoinsulinemia in these mice is possibly due to increased insulin clearance, as indicated by elevated C-peptide levels and normal pancreatic insulin levels indicating normal pancreatic function. Whether the unexpected low adiposity and normal insulin sensitivity despite the presence of glucose intolerance in the RenTgMK mice is secondary to hypoinsulinemia merits further investigation.

## Conflict of Interest Statement

The authors declare that the research was conducted in the absence of any commercial or financial relationships that could be construed as a potential conflict of interest.
